# Characteristics and fetal outcomes of pregnant women with hypertensive disorders in China: a 9-year national hospital-based cohort study

**DOI:** 10.1186/s12884-022-05260-3

**Published:** 2022-12-09

**Authors:** Yi Yang, Yanxia Xie, Mingrong Li, Yi Mu, Peiran Chen, Zheng Liu, Yanping Wang, Qi Li, Xiaohong Li, Li Dai, Juan Liang, Jun Zhu

**Affiliations:** 1 Department of obstetrics and gynecology intensive care unit Nursing, West China Second University Hospital, West China School of Nursing, Sichuan University, Ren Min South Road Section 3 No.17, Sichuan Chengdu, China; 2 Key Laboratory of Birth Defects and Related Diseases of Women and Children (Sichuan University), Ministry of Education, Ren Min South Road Section 3 No.17, Sichuan Chengdu, China; 3 National Office for Maternal and Child Health Surveillance of China, West China Second University Hospital, Sichuan University, Ren Min South Road Section 3 No.17, Chengdu Sichuan, China; 4 Department of Pediatric, West China Second University Hospital, Sichuan University, Ren Min South Road Section 3 No.17, Chengdu, Sichuan, China, Sichuan Chengdu, China; 5 Medical Big Data Center, Sichuan University, Ren Min South Road Section 3 No.17, Chengdu, Sichuan, China, Sichuan Chengdu, China; 6 Department of Obstetrics, West China Second University Hospital, Sichuan University, Ren Min South Road Section 3 No.17, Chengdu, Sichuan, China, Sichuan Chengdu, China

**Keywords:** Fetal outcome, Hypertensive disorder during pregnancy, Blood pressure, Management

## Abstract

**Background:**

Hypertensive disorders of pregnancy (HDP) are a growing concern and a challenge for maternity care providers as the prevalence of hypertension continues to increase. However, optimal management of HDP is unclear. Therefore, we aimed to explore the differences in adverse fetal outcomes among women with different subtypes of HDP and different blood pressure (BP) levels, to provide evidence-based management of HDP.

**Methods:**

We obtained data from China’s National Maternal Near-Miss Surveillance System from 2012 to 2020. Associations between BP management and adverse fetal outcomes, stratified by the four subtypes of HDP, were assessed using logistic regression analysis with a robust variance estimator.

**Results:**

For the period, a total of 393,353 pregnant women with HDP were included in the study; 8.51% had chronic hypertension, 2.27% had superimposed preeclampsia, 50.17% had preeclampsia or eclampsia, and 39.04% had gestational hypertension. The BP levels at delivery admission were mostly (61.14%) of non-severe stage 2 (systolic BP 140–159 mm Hg and/or diastolic BP 90–109 mm Hg) hypertension by American Heart Association classification. A high rate of adverse fetal outcomes was observed among women with HDP, especially among those aged < 20 or > 35 y or those diagnosed with superimposed preeclampsia. Compared with those with normal BP levels at delivery admission, we found an upward curve with increased risk among pregnant women with more severe BP levels, including the risk of preterm birth and small for gestational age (SGA) fetus. The odds ratios (ORs) of stillbirth, neonatal death, and low Apgar scores associated with severe stage 2 hypertension increased significantly. In addition, the association between BP at admission and fetal outcomes differed among women with varying HDP subtypes. Pregnant women with preeclampsia or eclampsia had an increased risk for preterm birth (adjusted OR [aOR], 1.33 [95% confidence interval {CI}, 1.07 ~ 1.65]) and SGA (aOR, 1.37 [95% CI, 1.10 ~ 1.71]) even when the admission BP was at stage 1 level.

**Conclusion:**

Greater attention should be paid to cases involving preeclampsia superimposed on chronic hypertension and pregnant women aged < 20 or > 35 y to mitigate the burden of adverse fetal outcomes caused by HDP.

**Supplementary Information:**

The online version contains supplementary material available at 10.1186/s12884-022-05260-3.

## Background

Hypertensive disorders of pregnancy (HDP) are a major cause of severe maternal and fetal illness that seriously threatens maternal and fetal health and are a leading cause of maternal death worldwide [1]. Additionally, affected women are at an increased risk of cardiovascular disease later in life, independent of traditional cardiovascular disease risks [2]. As the prevalence of hypertension continues to increase, pregnancies complicated by hypertensive disorders, including chronic hypertension, gestational hypertension, preeclampsia/eclampsia, and preeclampsia superimposed on chronic hypertension, are becoming a growing concern and challenge for obstetricians [3]. With the liberalisation of China’s “two-child” family planning policy in the past decade, the monthly mean proportion of pregnant women of advanced age (> 35 y) has increased from 8.5–13.5% [4]. Hence, the incidence of HDP has increased from 5.22% to 2011 to 6.40% in the period of 2014–2018 [5, 6], affecting almost 5.22–5.57% of pregnant women in China [6, 7]. The adverse maternal and fetal outcomes associated with HDP could greatly impact the general public health.

Several studies have focused on HDP management to optimise perinatal outcomes [8–11]; however, some are either limited to a specific HDP subtype, focused on a specific fetal outcome, or do not consider the blood pressure (BP) level. Nevertheless, the fact that control of BP within a specific range may reduce adverse maternal and infant outcomes in clinical practice [12] could be an important research direction. The CHIPS study found that the association between severe hypertension and serious maternal complications was observed only with a less tight control on BP (target diastolic BP [dBP]: 100 mmHg) [13]. In addition, studies on pregnant women with other comorbidities and a BP of > 160/105 mmHg suggested that BP be controlled to < 140/90 mmHg, and adhering to strict BP targets diligently will be beneficial for patients [12, 14, 15]. There is no unified recommendation for BP control goals in pregnant women with HDP due to the particularity of the pathophysiological changes during pregnancy and need to guarantee uterine-placental blood perfusion during antihypertensive treatment  [16–18]. Therefore, scientifically and precisely controlling BP can provide a longer time for the intrauterine growth of the fetus and also reduce adverse maternal and infant outcomes, which is a considerable challenge for obstetricians and researchers. Furthermore, because of larger sample size requirements, current studies still lack sufficient evidence and little is known regarding the association between BP management and rare adverse outcomes among patients with HDP, particularly in China.

This study aimed to use data from the National Maternal Near-Miss Surveillance System (NMNMSS) of China to identify the incidence of adverse fetal outcomes and to compare the association of their outcomes with BP management in four subtypes of HDP. Furthermore, this study aimed to explore the differences in adverse fetal outcomes among different subtypes of HDP with the goal of providing the evidence for the management of HDP through analysis of a cohort of pregnant women with HDP in China.

## Methods

### Study design and data sources

This national representative hospital-based study analysed pregnant women with singleton gestations using data from the NMNMSS of 438-member hospitals across China from 1 to 2012 to 31 December 2020. (We excluded three of the 441-member hospitals because they did not report data after 2012). Sociodemographic and obstetric information of pregnant and postpartum women visiting obstetric departments from admission to discharge was collected, and the data provided were de-identified.

### Study population and outcome definitions

We restricted our analysis to perinatal births that occurred at or after 28 weeks of gestation or where the birth weight of the baby was ≥ 1000 g, in accordance with the World Health Organization’s (WHO’s) definition [19]. The study population included women with hypertensive disorders who delivered during the study period. Women were categorised as having (i) chronic hypertension, (ii) superimposed preeclampsia, (iii) preeclampsia or eclampsia, and (iv) gestational hypertension, according to the guidelines of the American College of Obstetricians and Gynecologists [15]. Chronic hypertension was defined as hypertension (systolic BP [sBP] ≥ 140 mmHg or dBP ≥ 90 mmHg) before pregnancy or before 20 weeks of gestation. Superimposed preeclampsia was defined as chronic hypertension associated with preeclampsia. Preeclampsia was defined as hypertension and proteinuria after 20 weeks of gestation or hypertension plus involvement of at least one organ or system. Eclampsia was diagnosed when preeclampsia progressed to a convulsive phase. The diagnosis came from each member-hospital; the truthfulness and accuracy of the data were guaranteed through strict quality control in the process of data collection. The quality control includes five levels. Checking whether the diagnosis results reported to the NMNMSS by each hospital are consistent with those included in the medical records is the key quality control measure.

We defined gestational age according to the time of the last menstrual period or based on ultrasound assessment when the date of the last menstrual period was unknown. The perinatal outcomes of interest in this study were stillbirth, neonatal death, preterm birth (< 37 gestational weeks), SGA (birth weight less than the 10th percentile for gestational age), and low Apgar scores [20]. We calculated the stillbirth rate as the number of fetal losses after 28 weeks of gestation by the number of perinatal cases. We defined births before 37 weeks of gestation as preterm delivery and neonatal death as the death of a live-born infant up to 7 days after delivery. Apgar scores at 5 min were classified into two groups: abnormal (Apgar scores 1–6) and normal (Apgar scores 7–10). A baby was defined as being SGA if it weighed less than the 10th percentile based on gestational age-specific birth weight percentiles for male and female infants in China [21].

### Definitions of exposures

The BP data were obtained upon admission for delivery. BP was categorised using American Heart Association (AHA) 2017 guidelines [22] as normal BP [sBP < 120 mmHg and dBP < 80 mmHg], elevated BP [sBP 120–129 mmHg and dBP < 80 mmHg], stage 1 hypertension (sBP 130–139 mmHg, dBP 80–89 mmHg, or both), non-severe stage 2 hypertension (sBP 140–159 mm Hg, dBP 90–109 mm Hg, or both), and severe stage 2 hypertension (sBP ≥ 160 mmHg, dBP ≥ 110 mmHg, or both).

Maternal age was grouped as 15–24, 25–34, 35–44, and ≥ 45 y. In China, the government recommends five or more antenatal visits in rural areas and eight or more in urban areas; therefore, the number of antenatal care visits during pregnancy was categorised as none, one to four, five to seven, and eight or more. The usual definitions of the region (eastern, central, and western) and maternal educational status (illiteracy, primary school, middle school, high school, college or higher) were used as detailed elsewhere [10]. Diabetes is defined as including pre-pregnancy and gestational diabetes. Kidney disease is defined other than stones, cysts, and institutional abnormalities during pregnancy.

### Statistical analyses

Descriptive statistics were used to summarise the maternal characteristics and BP control level categories. Variables that were normally distributed are reported as means and standard deviations (SDs). Variables that were non-normally (categorical variables) distributed are reported as numbers and proportions.

We calculated the overall adverse outcome rate in women with HDP across the different year and age groups and identified the effect of BP management on perinatal outcomes across the four subtypes of HDP. The Mantel-Haenszel chi-square test by SPSS 23.0 was used to identify whether the rates have linear trends during 2012–2020. Usually, logistic regression analysis is regarded as an appropriate approach for analysing the rate of risk events such as preterm birth rate. Therefore, we performed a logistic regression analysis with a robust variance estimator to examine the association between different levels of BP and the proportion of adverse perinatal outcomes. We calculated adjusted odds ratios (aORs) and 95% confidence intervals (CIs) while adjusting for the clustering of births within hospitals, regions, antenatal care, marital status, age, parity, diabetes, kidney disease.

We defined *p* values of < 0.05 as significant. Analyses were performed using the Stata software (version 16.0; Stata Corp., College Station, TX, USA).

### Ethical approval

This study was approved by the ethics committee of the West China Second University Hospital (Protocol ID, 2012008).

## Results

A total of 393,353 pregnant women with HDP were included in the study (mean age [SD], 30.46 [5.57] y). Of these women, 33,471 (8.51%) had chronic hypertension, 8,941 (2.27%) had superimposed preeclampsia, 197,357 (50.17%) had preeclampsia or eclampsia, and 153,584 (39.04%) had gestational hypertension. The maternal characteristics of the pregnancies are presented in Table 1. As expected, pregnant women with HDP were older and had a shorter gestational age. A greater number of pregnant women with HDP were nulliparous (55.16%), lived in areas of central China (40.00%), and delivered through caesarean Sect. (67.48%). Most pregnant women with HDP had eight or more (55.63%) antenatal care visits. The BP levels at delivery admission were mostly distributed in the non-severe stage 2 hypertension (systolic BP 140–159 mm Hg and/or diastolic BP 90–109 mm Hg) group (61.14%). The application rate of magnesium sulphate was 52.04% in women with superimposed preeclampsia and 40.05% in those with preeclampsia or eclampsia. Maternal characteristics were similar among the different subtypes of HDP. However, compared with the other groups, those with superimposed preeclampsia had a higher proportion of adverse fetal outcomes (preterm birth, 45.15%; SGA, 25.47%; stillbirth, 40.8%; neonatal death, 8.9%; low Apgar score, 1.87%).

**Table 1:** Maternal characteristics by HDP status during pregnancy

	Total	Chronic hypertension	Superimposed preeclampsia	Preeclampsia or eclampsia	Gestational hypertension
**Age at delivery, years**	30.46 ± 5.57	31.89 ± 5.47	33.05 ± 5.55	30.17 ± 5.62	30.36 ± 5.44
**SBP, mmHg**	143.69 ± 16.23	141.59 ± 16.11	157.50 ± 21.48	147.17 ± 17.40	138.86 ± 12.30
**DBP, mmHg**	93.70 ± 11.55	92.35 ± 11.50	102.08 ± 13.90	95.92 ± 12.30	90.65 ± 9.34
**Blood pressure categories at birth admission, n (%)**					
Normal	10,360(2.63)	1230(3.67)	91(1.02)	4800(2.43)	4239(2.76)
Elevated	9564(2.43)	1105(3.30)	76(0.85)	3898(1.98)	4485(2.92)
Stage1	57,302(14.57)	6012(17.96)	632(7.07)	23,663(11.99)	26,995(17.58)
Non-severe stage2	240,487(61.14)	20,173(60.27)	3790(42.39)	108,501(54.98)	108,023(70.33)
Severe stage2	75,640(19.23)	4951(14.79)	4352(48.67)	56,495(28.63)	9842(6.41)
**Gestational age at delivery, weeks**	37.86 ± 2.34	37.88 ± 2.17	35.90 ± 3.11	37.38 ± 2.61	38.57 ± 1.63
**Antenantal care visits, n (%)**					
None	4485(1.14)	299(0.89)	154(1.72)	2826(1.43)	1206(0.79)
1–4	43,136(10.97)	2624(7.84)	1139(12.74)	25,346(12.84)	14,027(9.13)
5–7	111,479(28.34)	8071(24.11)	2351(26.29)	60,138(30.47)	40,919(26.64)
>=8	218,821(55.63)	21,193(63.32)	4567(51.08)	100,930(51.14)	92,131(59.99)
Unknown	15,432(3.92)	1284(3.84)	730(8.16)	8117(4.11)	5301(3.45)
**Region of China, n (%)**					
East	116,044(29.50)	11,044(33.00)	3185(35.62)	53,196(26.95)	48,619(31.66)
Central	159,289(40.50)	13,303(39.74)	3284(36.73)	81,160(41.12)	61,542(40.07)
West	118,020(30.00)	9124(27.26)	2472(27.65)	63,001(31.92)	43,423(28.27)
**Hospital level, n (%)**					
Unknown	18,593(4.73)	1320(3.94)	183(2.05)	10,400(5.27)	6690(4.36)
Level 1	16,999(4.32)	984(2.94)	193(2.16)	9133(4.63)	6689(4.36)
Level 2	152,599(38.79)	12,325(36.82)	2543(28.44)	74,195(37.96)	62,816(40.90)
Level 3	205,162(52.16)	18,842(56.29)	6022(67.35)	102,909(52.14)	77,389(50.39)
**Marital status, n (%)**					
Unmarried	5420(1.38)	349(1.04)	75(0.84)	2814(1.43)	2181(1.42)
Married	387,870(98.61)	33,115(98.94)	8863(99.13)	194,515(98.56)	151,377(98.56)
Unknown	63(0.02)	7(0.02)	3(0.03)	28(0.01)	25(0.02)
**Delivery method, n (%)**					
Vaginal	127,913(32.52)	11,464(34.25)	1484(16.60)	48,037(24.34)	66,928(43.58)
Caesarean section	265,440(67.48)	22,007(65.75)	7457(83.40)	149,320(75.66)	86,656(56.42)
**Parity, n (%)**					
Nulliparous	216,966(55.16)	16,469(49.20)	3685(41.21)	112,779(57.14)	84,033(54.71)
Multiparous	176,240(44.80)	16,993(50.77)	5255(58.77)	84,494(42.81)	69,498(45.25)
Unknown	147(0.04)	9(0.03)	1(0.01)	84(0.04)	53(0.03)
**Education, n (%)**					
No formal education	3201(0.81)	232(0.69)	89(1.00)	1752(0.89)	1128(0.73)
Primary school	15,892(4.04)	1181(3.53)	512(5.73)	8310(4.21)	5889(3.83)
Middle school	105,855(26.91)	7348(21.95)	2292(25.63)	55,731(28.24)	40,484(26.36)
High school	106,110(26.98)	8769(26.20)	2406(26.91)	54,761(27.75)	40,174(26.16)
College or higher	153,889(39.12)	15,133(45.21)	3462(38.72)	72,442(36.71)	62,852(40.92)
Unknown	8406(2.14)	808(2.41)	180(2.01)	4361(2.21)	3057(1.99)
**Application of magnesium sulfate, n (%)**	101,649 (25.84)	4036 (12.06)	4653(52.04)	79,044(40.05)	13,916(9.06)
**Diabetes, n (%)**	64,085(16.29)	7584(22.66)	2185(24.44)	28,132(14.25)	26,184(17.05)
**Kidney disease, n (%)**	4611(1.17)	427(1.28)	330(3.69)	2862(1.45)	992(0.65)
**Preterm delivery at < 37 weeks, n (%)**	70,162(17.85)	4882(14.60)	4024(45.15)	50,587(25.65)	10,669(6.95)
**SGA (< 10th percentile), n (%)**	67,643(17.22)	4016(12.02)	2266(25.47)	45,264(22.98)	16,097(10.49)
**Stillbirth, n (%)**	5375(1.37)	413(1.23)	365(4.08)	3734(1.89)	863(0.56)
**Neonatal death, n (%)**	1129(0.29)	72(0.22)	79(0.89)	785(0.40)	193(0.13)
**Low Apgar score, n (%)**	2797(0.73)	145(0.44)	158(1.87)	1937(1.01)	557(0.37)

**Fig. 1:**
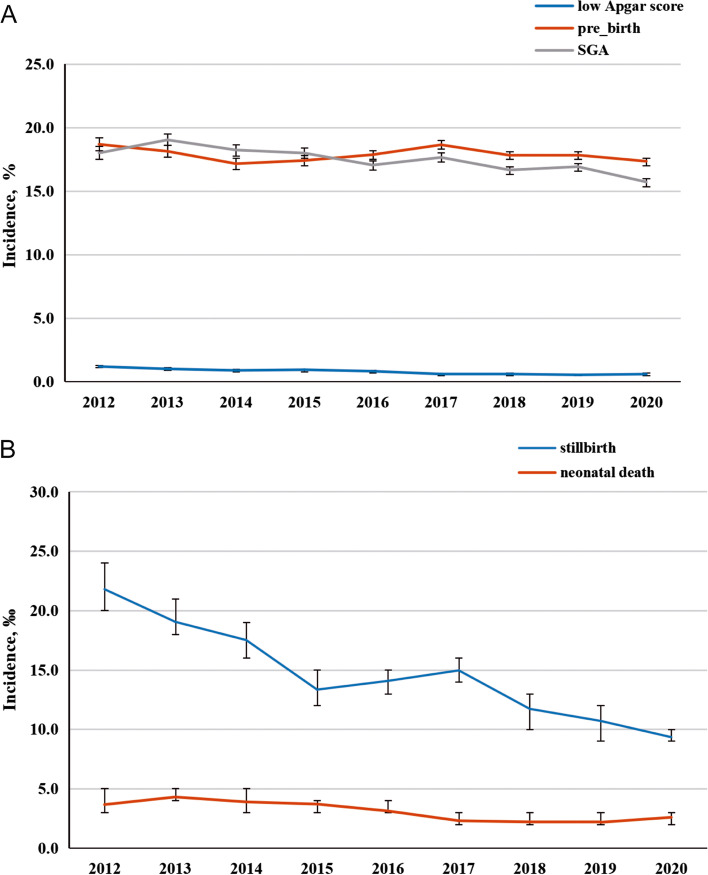
The secular trends and incidence of adverse fetal outcome among HDP pregnant women during 2012–2020. HDP, hypertension disorders during pregnancy. **a** preterm birth, SGA, and low Apgar score; **b** neonatal death, and stillbirth

**Fig. 2:**
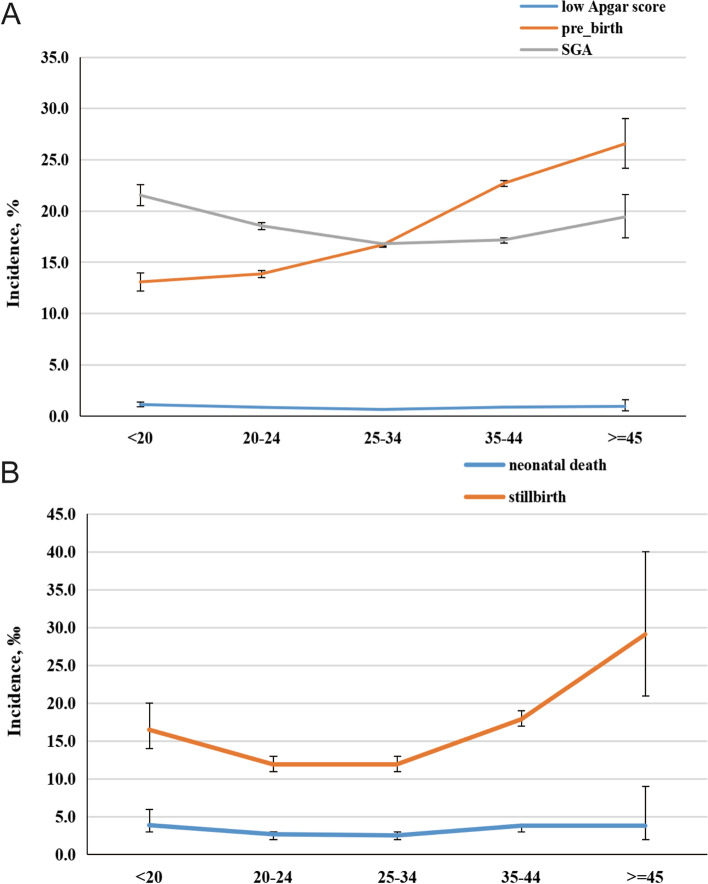
The incidence of adverse fetal outcome across different age group among HDP pregnant women during 2012–2020. HDP, hypertension disorders during pregnancy. **a** preterm birth, SGA, and low Apgar score; **b** neonatal death, and stillbirth

The temporal prevalence of adverse fetal outcomes among pregnant women with HDP is depicted in Fig. 1. Overall, the incidence of adverse fetal outcomes declined steadily from 2012 to 2020 in China. For instance, the incidence of preterm birth decreased from 18.7 to 17.3% (*p* < 0.001), the incidence of SGA decreased from 18.0 to 15.7% (*p* < 0.001), and a decrease in stillbirths (*p* < 0.001), neonatal deaths (*p* < 0.001), and low Apgar scores (*p* < 0.001) was observed. The association between maternal age and the incidence of adverse fetal outcomes is depicted in Fig. 2. The incidence of SGA and a low Apgar score was the highest at < 20 y of age, while the incidence of preterm birth increased from 13.1% at < 20 y to 26.5% at ≥ 45 y of maternal age. A similar trend was found with the stillbirth rate among the maternal age groups. Additionally, the incidence of neonatal death was relatively high in those aged < 20, 35–44, and ≥ 45 y.

According to the logistic regression analysis, the associations between the BP level at admission and risk of adverse fetal outcomes are depicted in Table 2. Pregnant women with severe stage 2 hypertension had higher rates of preterm birth, stillbirth, SGA, neonatal death, and low Apgar scores than other pregnant women. The BP level at delivery admission was strongly associated with adverse pregnancy outcomes in women with HDP. Compared with those of normal BP level, we found an upward curve with increased risk among pregnant women with more severe BP level at delivery admission, including risk of preterm birth (elevated: aOR, 1.08 [95% CI, 0.95 ~ 1.23], stage 1: aOR, 1.09 [95% CI, 0.97 ~ 1.23], non-severe stage 2: aOR, 1.67 [95% CI, 1.46 ~ 1.91], severe stage 2: aOR, 5.44 [95% CI, 4.70 ~ 6.28]) and SGA (elevated: aOR, 1.01 [95% CI, 0.87 ~ 1.16], stage 1: aOR, 1.21 [95% CI, 1.07 ~ 1.38], non-severe stage 2: aOR, 1.55 [95% CI, 1.34 ~ 1.79], severe stage 2: aOR, 3.20 [95% CI, 2.74 ~ 3.73]). The ORs of stillbirth (aOR, 2.55 [95% CI, 1.96 ~ 3.32]), neonatal death (aOR, 2.24 [95% CI, 1.42 ~ 3.54]), and low Apgar score (aOR, 2.92 [95% CI, 2.20 ~ 3.89]) associated with severe stage 2 hypertension increased greatly.

**Table 2:** Fetal adverse outcome by severity of HDP during pregnancy (Total)

	Normal	Elevated	Stage1	Non-severe stage2	Severe stage2
**Preterm delivery at < 37 weeks**					
Frequency, n (%)	1078(10.41)	1042(10.90)	6076(10.61)	34,128(14.20)	27,838(30.86)
OR (95% CI)	ref.	1.05(0.93 ~ 1.19)	1.02(0.91 ~ 1.14)	1.42(1.25 ~ 1.62)	5.02(4.36 ~ 5.79)
aOR (95% CI)	ref.	1.08(0.95 ~ 1.23)	1.09(0.97 ~ 1.23)	1.67(1.46 ~ 1.91)	5.44(4.70 ~ 6.28)
**SGA (< 10th percentile)**					
Frequency, n (%)	1179(11.40)	1031(10.80)	7195(12.57)	36,925(15.37)	21,313(28.27)
OR (95% CI)	ref.	0.94(0.83 ~ 1.07)	1.12(1.00 ~ 1.25)	1.41(1.24 ~ 1.61)	3.06(2.66 ~ 3.53)
aOR (95% CI)	ref.	1.01(0.87 ~ 1.16)	1.21(1.07 ~ 1.38)	1.55(1.34 ~ 1.79)	3.20(2.74 ~ 3.73)
**Stillbirth**					
Frequency, n (%)	98(0.95)	78(0.82)	462(0.81)	2495(1.04)	2242(2.96)
OR (95% CI)	ref.	0.86(0.64 ~ 1.17)	0.85(0.68 ~ 1.06)	1.10(0.86 ~ 1.41)	3.20(2.51 ~ 4.08)
aOR (95% CI)	ref.	0.97(0.71 ~ 1.33)	0.97(0.77 ~ 1.23)	1.20(0.92 ~ 1.56)	2.55(1.96 ~ 3.32)
**Neonatal death**					
Frequency, n (%)	25(0.24)	18(0.19)	101(0.18)	501(0.21)	484(0.64)
OR (95% CI)	ref.	0.78(0.46 ~ 1.33)	0.73(0.45 ~ 1.18)	0.86(0.55 ~ 1.34)	2.66(1.71 ~ 4.15)
aOR (95% CI)	ref.	0.85(0.50 ~ 1.44)	0.81(0.51 ~ 1.29)	0.98(0.62 ~ 1.54)	2.24(1.42 ~ 3.54)
**low Apgar score**					
Frequency, n (%)	51(0.50)	34(0.36)	228(0.40)	1243(0.53)	1241(1.71)
OR (95% CI)	ref.	0.72(0.47 ~ 1.10)	0.81(0.60 ~ 1.10)	1.06(0.78 ~ 1.42)	3.48(2.55 ~ 4.73)
aOR (95% CI)	ref.	0.77(0.51 ~ 1.14)	0.88(0.67 ~ 1.17)	1.10(0.83 ~ 1.45)	2.92(2.20 ~ 3.89)

In addition, our findings on the association between BP on admission and fetal outcomes differed among women with varying subtypes of HDP (Supplement Table 1). Overall, pregnant women with superimposed preeclampsia, preeclampsia, or eclampsia had a higher rate of adverse outcomes even with the same BP level at admission. Compared with pregnant women with other HDP subtypes, those with preeclampsia or eclampsia had an increased risk of preterm birth (aOR, 1.33 [95% CI, 1.07 ~ 1.65]) and SGA (aOR, 1.37 [95% CI, 1.10 ~ 1.71]) even when the admission BP level was stage 1 (sBP 130–139 mm Hg, dBP 80–89 mm Hg, or both). Additionally, we observed that severe stage 2 hypertension was strongly associated with stillbirth in pregnant women with preeclampsia, eclampsia (aOR, 2.26 [95% CI, 1.50 ~ 3.41]), or gestational hypertension (aOR, 2.21 [95% CI, 1.31 ~ 3.71]). Moreover, the association between severe stage 2 hypertension and neonatal death was observed only in pregnant women with preeclampsia or eclampsia (aOR, 2.14 [95% CI, 1.15 ~ 3.99]). The risks of preterm birth were significantly decreased in those with stage 1 BP levels or of stillbirth in those with stage 1 and non-severe stage 2 BP levels among women diagnosed with chronic hypertension. For women with gestational hypertension, there was a significant decrease in the risk of preterm birth among those with stage 1 BP levels. Additionally, we observed that the risk of neonatal death was significantly decreased in those with stage 1 and non-severe stage 2 BP levels among pregnant women with superimposed preeclampsia.

## Discussion

This large cohort study revealed a high risk of adverse fetal outcomes among pregnant women with HDP in China, especially among pregnant women aged < 20 and > 35 y. We also found an association between BP at delivery admission and fetal outcomes, which differed among women with different subtypes of HDP. Two of the adverse fetal outcomes that we observed (preterm birth and SGA) were associated with the BP level at delivery admission; regardless of the HDP subtype (pregnant women with preeclampsia or eclampsia had an increased risk of preterm birth and SGA even when the admission BP level was stage 1). However, for some adverse fetal outcomes, including stillbirth, neonatal death, and low Apgar score, increased risks were found among those with chronic hypertension, preeclampsia, eclampsia, or gestational hypertension who had a severe stage 2 BP level at admission compared with pregnant women of the same subtype with a normal BP level.

The effect of HDP on fetal outcomes varies among populations. A Taiwanese (China) study found that HDP caused 26.2% of preterm births [23], while a recent study from Japan showed that women with HDP have 21.2% of preterm births [24]. Our study found that preterm birth rates in women with HDP (17.85%) might not be higher than the rates found in the above-mentioned studies. Moreover, the preterm birth rate of women with chronic hypertension is similar in China and the United States (14.6% vs. 14.1%) [8], while the incidence of SGA among women follows an opposite trend. The incidence of SGA was 17.22%, 11.3%, and 13.8% in our study, Taiwan, and Japan, respectively. The differences might be due to the variations in age distribution, socioeconomic backgrounds, ethnicity, or different SGA definition. Notably, most of these studies typically consider pregnant women with HDP. Few studies have compared the adverse fetal outcomes from the different subtypes included in this group; studies with a larger sample size are needed for substantial results. In addition, identifying differences in adverse fetal outcomes between pregnant women with and without HDP is generally easier; however, such results may have limited clinical significance. When we enrolled pregnant women with HDP in this study and compared their association with all the subtypes of HDP, we found that those with superimposed preeclampsia had a higher proportion of adverse fetal outcomes. Previous studies have reported similar results [11, 25–27]; preterm birth was significantly more frequent among pregnant women with superimposed preeclampsia.

Moreover, our findings support the fact that the association between the BP level at delivery admission and fetal outcomes differed among women with varying subtypes of HDP, which has not been observed in previous studies due to the incomplete inclusion of diseases. These results indicated that the fetal outcomes of pregnant women with different HDP subtypes or different BP levels of the same subtype were significantly different, suggesting that a differential management of pregnant women with HDP is required.

The molecular and pathophysiological mechanisms of HDP, especially preeclampsia, are largely unknown, although the cause is likely a combination of and interaction between factors from both maternal and placental pathways [21, 28]. When the rate of uteroplacental flow is chronically reduced, a strong direct linear correlation exists between the rate of mean uteroplacental blood flow and placental weight. The placenta has the ability to control fetal growth [29] and precisely explains the phenomenon found in this study that the higher the BP level, the more severe the adverse fetal outcomes will be. The guidelines for HDP management in China recommend the administration of intravenous magnesium sulphate for preeclampsia and severe hypertension or hypertension with neurologic symptoms; however, this injection rate was only 52.04% in women with superimposed preeclampsia and 40.05% in women with preeclampsia or eclampsia in our study [30]. This finding indicates the problem of suboptimal management in women with HDP throughout pregnancy, which caused nearly 80% of women to have a BP of > 140/90 mmHg at delivery admission and 24% of them to have a BP of > 160/110 mmHg. In addition, the universal ‘two-child’ policy has been associated with changes in maternal characteristics (more likely for those aged > 35 y) [4]. Simultaneously, the prevalence of chronic hypertension in this age group increases with age [31], and the elasticity of blood vessels decreases accordingly [32]. These studies indicate that more attention should be paid to the management during pregnancy in women of advanced maternal age diagnosed with HDP. In addition, although the adverse fetal outcomes of women with HDP aged < 20 y should also be a concern for obstetricians.

The management of HDP has not yet been unified [16–18, 22, 30]. In general, maintaining a goal of a BP of < 160/110 mmHg [33, 34] has been recommended. In addition, the ACC/AHA recommends that in women with a risk of hypertensive crises (severe preeclampsia or eclampsia), sBP should be reduced to < 140 mmHg during the first hour of treatment [22]. The International Society for the Study of Hypertension in Pregnancy recommends that a BP of ≥ 140/90 mmHg (or ≥ 135/85 mmHg at home) should be treated with a goal to attain a BP of 110–140/85 mmHg [16]. Canada’s Pregnancy Guidelines on Hypertension recommend antihypertensive therapy for non-severe hypertension (sBP ≥ 140 mmHg or dBP ≥ 90 mmHg) with a target of dBP measuring 85 mmHg [17]. The guidelines involved in the management of pregnant women with HDP recommend that pregnant women without risk factors should be treated with a goal to attain a BP of 130–140/80–90 mmHg, and those with target organ damage should be treated with a goal BP of 130–135/80–85 mmHg [30]. These variations arise from limited evidence to drive clinical practice and to reflect the reality that many aspects of the guidelines emanate from expert opinions rather than high-quality evidence. Most current guidelines manage HDP in a broad category rather than in subtypes. However, these four subtypes may have different pathological mechanisms and, therefore, may play different roles in fetal outcomes [10, 35]. Our findings support the hypothesis that the association between BP level at admission and fetal outcomes differs among women with varying subtypes of HDP. This study is one of the few to examine each subgroup of HDP and to provide evidence to support current guidelines for the management of HDP. We suggest that BP management goals based on different HDP subtypes and appropriately strict BP targets should be considered in future guidelines.

The strength of our study is that we had a large sample size of 393,353 pregnant women, which guarantees that we assessed the risk caused by BP management conferred by the four subtypes of HDP separately. Therefore, our results are reliable and of great significance in clinical practice. One major limitation of our study was the difficulty in acquiring and adjusting for medication information. However, we calculated the risk of adverse fetal outcomes by adjusting for other factors and consequently, the results are likely to be realistic. Another limitation is that we could not obtain the gestational age of the enrolled women at the time of diagnosis, which would have optimised our results to consider the duration of HDP. Finally, we calculated the gestational age using the last menstrual period, whereas early pregnancy ultrasound is now considered the gold standard.

## Conclusion

In summary, our study has several important implications. HDP increases the risk of adverse fetal outcomes in mothers of younger or advanced age. Considering that HDP is a dynamic process, greater attention should be paid to the management of pregnancy in patients with hypertension, and individualised management of hypertension should be encouraged. Moreover, as the risk varies among the four HDP subtypes, different management strategies should be implemented in the future.

## Supplementary Information

**Additional file 1: Supplement Table 1.** Fetal adverse outcome by severity of HDP during pregnancy across four subtypes.

## Data Availability

The datasets generated and/or analysed during the current study are not publicly available due to the terms of our contract with the Chinese National Health Commission but are available from the corresponding author on reasonable request.

## Abbreviations

HDP: Hypertensive disorder during pregnancy
BP: Blood pressure
NMNMSS: China’s National Maternal Near Miss Surveillance System
ACC/AHA: American College of Cardiology / American Heart Association
