# Supplementary material for: Characteristics and fetal outcomes of pregnant women with hypertensive disorders in China: a 9-year national hospital-based cohort study
Source: BMC Pregnancy Childbirth. 2022 Dec 9;22:924. doi: 10.1186/s12884-022-05260-3 (PMC9733350; doi:10.1186/s12884-022-05260-3)
Supplement: Supplementary file 1 — Additional file 1: Supplement Table 1. Fetal adverse outcome by severity of HDP during pregnancy across four subtypes. [file 12884_2022_5260_MOESM1_ESM.docx]

| **Supplement Table1.** Fetal adverse outcome by severity of HDP during pregnancy across four subtypes. | | | | | |
| --- | --- | --- | --- | --- | --- |
|  | Normal | Elevated | Stage1 | Non-severe stage2 | Severe stage2 |
| **Chronic hypertension** | | | | | |
| ***Preterm delivery at <37 weeks*** |  |  |  |  |  |
| *Frequency, n (%)* | 152(12.39) | 122(11.06) | 555(9.24) | 2441(12.11) | 1612(32.62) |
| *OR (95% CI)* | ref. | 0.88(0.68~1.13) | 0.72(0.59~0.88) | 0.97(0.81~1.18) | 3.42(2.76~4.25) |
| *aOR (95% CI)* | ref. | 0.93(0.73~1.19) | 0.78(0.63~0.95) | 1.10(0.91~1.33) | 3.55(2.85~4.42) |
| ***SGA (<10th percentile)*** |  |  |  |  |  |
| *Frequency, n (%)* | 108(8.82) | 73(6.62) | 546(9.09) | 2247(11.15) | 1042(21.14) |
| *OR (95% CI)* | ref. | 0.73(0.54~0.99） | 1.03(0.84~1.27) | 1.30(1.06~1.59) | 2.77(2.19~3.50) |
| *aOR (95% CI)* | ref. | 0.78(0.58~1.05） | 1.09(0.88~1.35) | 1.30(1.06~1.60） | 2.65(2.09~3.36) |
| ***Stillbirth*** |  |  |  |  |  |
| *Frequency, n (%)* | 21(1.71) | 10(0.90) | 46(0.77) | 185(0.92) | 151(3.05) |
| *OR (95% CI)* | ref. | 0.53(0.24~1.13) | 0.44(0.27~0.72) | 0.53(0.37~0.77) | 1.81(1.26~2.60) |
| *aOR (95% CI)* | ref. | 0.61(0.29~1.29) | 0.51(0.31~0.83) | 0.52(0.35~0.79) | 1.23(0.80~1.89) |
| ***Neonatal death*** |  |  |  |  |  |
| *Frequency, n (%)* | 1(0.08) | 2(0.18) | 9(0.15) | 29(0.14) | 31(0.63) |
| *OR (95% CI)* | ref. | 2.23(0.13~37.30) | 1.84(0.22~15.10) | 1.77(0.26~12.10) | 7.74(1.04~57.42) |
| *aOR (95% CI)* | ref. | 22.37(0.13~43.26) | 2.03(0.25~16.73) | 2.04(0.29~14.17) | 7.36(0.98~55.08) |
| ***low Apgar score*** |  |  |  |  |  |
| *Frequency, n (%)* | 1(0.08) | 0(0.00) | 15(0.26) | 67(0.34) | 62(1.31) |
| *OR (95% CI)* | ref. | - | 3.04(0.38~24.42) | 4.07(0.55~30.12) | 15.95(3.12~119.25) |
| *aOR (95% CI)* | ref. | - | 3.29(0.41~26.36) | 4.12(0.56~30.48) | 13.79(1.85~102.70) |
| ***Superimposed preeclampsia*** | | | | | |
| ***Preterm delivery at <37 weeks*** |  |  |  |  |  |
| *Frequency, n (%)* | 24(26.37) | 18(23.68) | 168(26.58) | 1322(34.94) | 2492(57.55) |
| *OR (95% CI)* | ref. | 0.87(0.43~1.73) | 1.01(0.61~1.69) | 1.50(0.91~2.46) | 3.79(2.30~6.24) |
| *aOR (95% CI)* | ref. | 0.95(0.46~1.94) | 1.10(0.66~1.86) | 1.74(1.05~2.88) | 3.90(2.34~6.50) |
| ***SGA (<10th percentile)*** |  |  |  |  |  |
| *Frequency, n (%)* | 20(21.98) | 11(14.47) | 98(15.51) | 791(20.94) | 1346(31.16) |
| *OR (95% CI)* | ref. | 0.60(0.28~1.30) | 0.65(0.39~1.09) | 0.94(0.56~1.57) | 1.61(0.93~2.77) |
| *aOR (95% CI)* | ref. | 0.65(0.31~1.36) | 0.69(0.41~1.14) | 1.01(0.61~1.68) | 1.62(0.95~2.76) |
| ***Stillbirth*** |  |  |  |  |  |
| *Frequency, n (%)* | 2(2.20) | 0(0.00) | 15(2.37) | 95(2.51) | 253(5.81) |
| *OR (95% CI)* | ref. | - | 1.08(0.25~4.62) | 1.14(0.30~4.37) | 2.75(0.76~9.97) |
| *aOR (95% CI)* | ref. | - | 1.24(0.30~5.05) | 1.29(0.37~4.48) | 2.23(0.66~7.52) |
| ***Neonatal death*** |  |  |  |  |  |
| *Frequency, n (%)* | 2(2.20) | 0(0.00) | 2(0.32) | 18(0.48) | 57(1.32) |
| *OR (95% CI)* | ref. | - | 0.14(0.02~1.00) | 0.21(0.05~0.93) | 0.59(0.14~2.55) |
| *aOR (95% CI)* | ref. | - | 0.12(0.02~0.90) | 0.19(0.04~0.88) | 0.39(0.09~1.83) |
| ***low Apgar score*** |  |  |  |  |  |
| *Frequency, n (%)* | 1(1.12) | 1(1.32) | 2(0.33) | 37(1.02) | 117(2.90) |
| *OR (95% CI)* | ref. | 1.17(0.07~19.60) | 0.29(0.03~3.17) | 0.90(0.12~6.74) | 2.63(0.36~19.14) |
| *aOR (95% CI)* | ref. | 1.31(0.07~23.59) | 0.30(0.03~3.52) | 0.96(0.12~7.70) | 2.17(0.27~17.16) |
| **Preeclampsia or eclampsia** | | | | | |
| ***Preterm delivery at <37 weeks*** |  |  |  |  |  |
| *Frequency, n (%)* | 595(12.40) | 564(14.48) | 3720(15.72) | 23361(21.54) | 22347(39.62) |
| *OR (95% CI)* | ref. | 1.20(0.96~1.49) | 1.32(1.07~1.62) | 1.94(1.54~2.44) | 4.63(3.65~5.88) |
| *aOR (95% CI)* | ref. | 1.18(0.93~1.49) | 1.33(1.07~1.65) | 2.15(1.69~2.73) | 4.84(3.78~6.20) |
| ***SGA (<10th percentile)*** |  |  |  |  |  |
| *Frequency, n (%)* | 664(13.84) | 567(14.58) | 4063(17.19) | 22602(20.86) | 17368(30.84) |
| *OR (95% CI)* | ref. | 1.06(0.85~1.32) | 1.29(1.05~1.59) | 1.64(1.31~2.06) | 2.77(2.20~3.50) |
| *aOR (95% CI)* | ref. | 1.10(0.87~1.40) | 1.37(1.10~1.71) | 1.80(1.40~2.30) | 2.93(2.27~3.77) |
| ***Stillbirth*** |  |  |  |  |  |
| *Frequency, n (%)* | 56(1.17) | 43(1.10) | 281(1.19) | 1639(1.51) | 1715(3.04) |
| *OR (95% CI)* | ref. | 0.94(0.65~1.36) | 1.02(0.73~1.42) | 1.30(0.91~1.85) | 2.65(1.86~3.78) |
| *aOR (95% CI)* | ref. | 1.07(0.71~1.60) | 1.15(0.79~1.67) | 1.42(0.94~2.13) | 2.26(1.50~3.41) |
| ***Neonatal death*** |  |  |  |  |  |
| *Frequency, n (%)* | 14(0.29) | 7(0.18) | 61(0.26) | 327(0.30) | 376(0.67) |
| *OR (95% CI)* | ref. | 0.62(0.29~1.31) | 0.88(0.46~1.71) | 1.03(0.56~1.89) | 2.29(1.24~4.23) |
| *aOR (95% CI)* | ref. | 0.69(0.32~1.47) | 1.00(0.53~1.90) | 1.20(0.65~2.21) | 2.14(1.15~3.99) |
| ***low Apgar score*** |  |  |  |  |  |
| *Frequency, n (%)* | 33(0.70) | 18(0.47) | 139(0.60) | 755(0.71) | 992(1.84) |
| *OR (95% CI)* | ref. | 0.67(0.39~1.14) | 0.86(0.59~1.24) | 1.02(0.71~1.46) | 2.66(1.84~3.85) |
| *aOR (95% CI)* | ref. | 0.76(0.45~1.27) | 0.98(0.69~1.39) | 1.15(0.83~1.61) | 2.56(1.82~3.60) |
| **Gestational hypertension** | | | | | |
| ***Preterm delivery at <37 weeks*** |  |  |  |  |  |
| *Frequency, n (%)* | 307(7.25) | 338(7.54) | 1633(6.05) | 7004(6.49) | 1387(14.10) |
| *OR (95% CI)* | ref. | 1.04(0.91~1.20) | 0.82(0.74~0.92) | 0.89(0.80~0.99) | 2.10(1.84~2.40) |
| *aOR (95% CI)* | ref. | 1.05(0.91~1.21) | 0.86(0.77~0.96) | 0.98(0.88~1.10) | 2.20(1.91~2.52） |
| ***SGA(<10th percentile)*** |  |  |  |  |  |
| *Frequency, n (%)* | 387(9.15) | 380(8.49) | 2488(9.22) | 11285(10.45) | 1557(15.85) |
| *OR (95% CI)* | ref. | 0.92(0.79~1.07) | 1.01(0.89~1.14) | 1.16(1.01~1.33) | 1.87(1.60~2.19) |
| *aOR (95% CI)* | ref. | 0.95(0.82~1.10) | 1.05(0.93~1.18) | 1.17(1.04~1.31) | 1.83(1.61~2.09) |
| ***Stillbirth*** |  |  |  |  |  |
| *Frequency, n (%)* | 19(0.45) | 25(0.56) | 120(0.44) | 576(0.53) | 123(1.25) |
| *OR (95% CI)* | ref. | 1.24(0.65~2.40) | 0.99(0.59~1.67) | 1.19(0.71~2.01) | 2.81(1.63~4.84) |
| *aOR (95% CI)* | ref. | 1.27(0.67~2.44) | 1.07(0.65~1.77) | 1.16(0.70~1.91) | 2.21(1.31~3.71) |
| ***Neonatal death*** |  |  |  |  |  |
| *Frequency, n (%)* | 8(0.19) | 9(0.20) | 29(0.11) | 127(0.12) | 20(0.20) |
| *OR (95% CI)* | ref. | 1.06(0.50~2.28) | 0.57(0.30~1.08) | 0.62(0.33~1.16) | 1.08(0.54~2.15) |
| *aOR (95% CI)* | ref. | 1.08(0.51~2.31) | 0.64(0.34~1.21) | 0.66(0.35~1.24) | 0.95(0.48~1.89) |
| ***low Apgar score*** |  |  |  |  |  |
| *Frequency, n (%)* | 16(0.38) | 15(0.34) | 72(0.27) | 384(0.36) | 70(0.73) |
| *OR (95% CI)* | ref. | 0.89(0.47~1.67) | 0.71(0.44~1.15) | 0.95(0.58~1.53) | 1.92(1.12~3.26) |
